# GABA_A_ receptor-expressing neurons promote consumption in *Drosophila melanogaster*

**DOI:** 10.1371/journal.pone.0175177

**Published:** 2017-03-31

**Authors:** Samantha K. Cheung, Kristin Scott

**Affiliations:** 1 Department of Molecular and Cell Biology, University of California, Berkeley, California, United States of America; 2 Helen Wills Neuroscience Institute, University of California, Berkeley, California, United States of America; AgroParisTech, FRANCE

## Abstract

Feeding decisions are highly plastic and bidirectionally regulated by neurons that either promote or inhibit feeding. In *Drosophila melanogaster*, recent studies have identified four GABAergic interneurons that act as critical brakes to prevent incessant feeding. These GABAergic neurons may inhibit target neurons that drive consumption. Here, we tested this hypothesis by examining GABA receptors and neurons that promote consumption. We find that Resistance to dieldrin (RDL), a GABA_A_ type receptor, is required for proper control of ingestion. Knockdown of *Rdl* in a subset of neurons causes overconsumption of tastants. Acute activation of these neurons is sufficient to drive consumption of appetitive substances and non-appetitive substances and acute silencing of these neurons decreases consumption. Taken together, these studies identify GABA_A_ receptor-expressing neurons that promote *Drosophila* ingestive behavior and provide insight into feeding regulation.

## Introduction

The ability to adjust feeding behaviors in different environments and contexts is essential for an animal to survive. Homeostatic mechanisms that regulate food intake balance caloric consumption with energy expenditure, which influences the health, fitness, and body weight of an organism [1]. Homeostatic regulation requires the integration of internal nutritional state, external sensory signals, and associations with past food-related experiences.

The fruit fly, *Drosophila melanogaster*, is an excellent model system to examine the neural circuits underlying the regulation of feeding behaviors; the fly brain contains a million fold fewer neurons compared to the human brain[2] and powerful genetic tools enable the manipulation of specific neurons. These features make the fly brain a tractable system for uncovering the neural circuitry for feeding regulation. The circuit principles uncovered in *Drosophila* may be shared throughout the animal kingdom.

The fly has stereotypical feeding behaviors involving a series of feeding subprograms. Feeding behavior initially involves detection of a potential food source with the legs or proboscis, which contain taste receptors neurons that allow the fly to make an evaluation before attempting consumption [3]. Detection of sugars drives feeding initiation, whereas detection of bitter compounds promotes rejection. Once a palatable food source is detected, a fly initiates feeding by using the proboscis extension response (PER). As the fly consumes, it detects appetitive substances with taste neurons of the pharynx or internal mouthparts [3,4]. The fly continues to ingest until internal cues signal satiety.

Some taste-responsive neural components of these feeding subprograms have been uncovered. Distinct classes of gustatory sensory neurons detect either water, sugars, bitter compounds, or pheromones [5–8]. Gustatory neurons on the legs, proboscis labellum, and mouthparts send projections to non-overlapping regions in the subesophageal zone (SEZ), a taste-processing region in the brain [6,9]. Two sucrose-responsive classes of local interneurons in the SEZ play a role in sucrose consumption: feeding neurons (FDG) respond to sucrose detection on the proboscis to promote initial consumption of sucrose [10], while ingestion neurons (IN1), respond to sucrose detection in the pharynx to promote sustained sucrose consumption in hungry flies [11]. In addition, candidate second-order neurons that send their projections to the antennal mechanosensory and motor center (AMMC) are sucrose responsive and trigger sucrose acceptance [12]. Finally, SEZ motor neurons that are necessary for feeding initiation and consumption have been identified [13,14].

How are the circuits that regulate feeding modulated by internal state? Although several studies have identified modulatory neurons that promote feeding when the fly is food-deprived [15,16], very few have examined how feeding is terminated when the fly is sated. A recent study identified a set of four interneurons, named DSOG1, that play an essential role in inhibiting consumption [17]. These neurons are GABAergic neurons that send wide arborizations throughout the SEZ. Remarkably, flies with silenced DSOG1 neurons consume vast quantities of appetitive and non-appetitive substances, even under sated conditions. This suggests that DSOG1 acts to inhibit indiscriminate consumption. To examine how DSOG1 neurons suppress consumption, we searched for downstream neurons that might receive the GABA signals from DSOG1. Here, we characterize neurons that express RDL, a GABA_A_ type receptor, that promote consumption, providing insight into the circuits that dynamically regulate feeding.

## Results

### *Rdl* is required for wild-type feeding behavior

**Fig 1:**
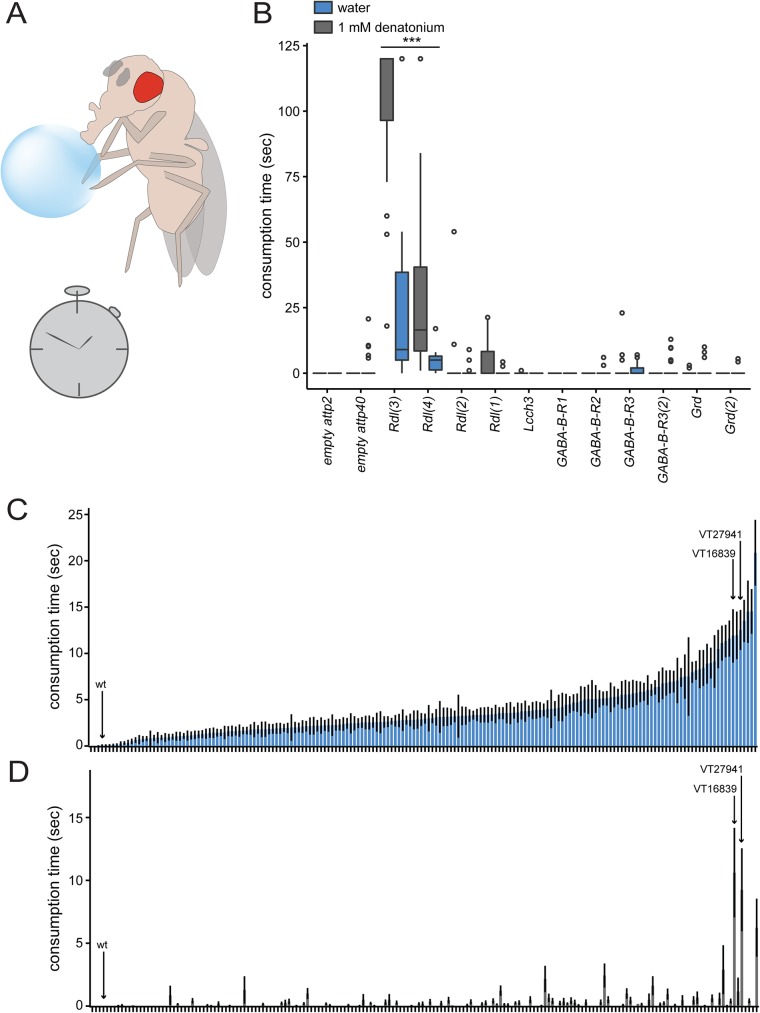
*Rdl* modulates consumption regulation. **A)** Diagram illustrating the temporal consumption assay. The time individual flies consumed various substances was recorded. **B)** GABAergic receptors were tested for their role in water and bitter (1 mM denatonium) consumption using RNAi to knock down expression pan-neuronally with *nSyb-Gal4; UAS-Dcr2* flies. Box plots show median consumption time for water consumption and 1 mM denatonium in fed flies. Different RNAi lines (different sequences targeting the same receptor) are denoted in (#). Knockdown of *Rdl* resulted in overconsumption of water and bitter. Kruskal-Wallis test, Dunn’s post-hoc; ***p<0.0001 indicates significance to *UAS-empty RNAi* controls for water or bitter consumption, as appropriate; n = 10-33/genotype. **C)** Screen for water overconsumption candidates (mean ± SEM); Gal4 lines were tested for their role in water consumption through RNAi of *Rdl*. Bars show consumption times (mean ± SEM) for water in fed flies. On the X-axis, each column represents a Gal4 line or wild-type (wt) lines. Gal4 lines were ordered from least to most consumption time. Arrows point to wild-type (IsoD1), *VT16839-Gal4*, and *VT27941-Gal4*. n = 10-22/genotype. **D)** Screen for bitter overconsumption candidates; Gal4 lines were tested for their role in bitter consumption through RNAi of *Rdl*. Bars show consumption times (mean ± SEM) for 1 mM denatonium in fed flies. On the X-axis, each column represents a Gal4 line or wild-type (wt). Gal4 lines are ordered corresponding to Fig 1C. Arrows point to wild-type (IsoD1), *VT16839-Gal4*, and *VT27941-Gal4*. The majority of Gal4 lines did not show consumption of denatonium. n = 10-22/genotype.

Since DSOG1 neurons require GABA production to inhibit consumption [17], we reasoned that they may inhibit GABA-receptor expressing neurons that are sufficient to drive ingestive behaviors. We therefore tested whether reducing expression of GABA receptors influenced feeding. There are five GABAergic receptors in *Drosophila*: a heteromultimeric cationic channel composed of the subunits GABA and glycine-like receptor of *Drosophila* (GRD) and ligand-gated chloride channel homolog 3 (LCCH3) [18]; a GABA_A_ type receptor (RDL) [19]; and 3 GABA_B_ receptors (GABA_B_ R1, GABA_B_ R2, GABA_B_ R3) [20]. To determine which GABAergic receptor(s) regulate consumption, we knocked down expression of the existing GABAergic receptors pan-neuronally using *nSyb-Gal4*[21] to drive RNA interference[22] and measured the effect on the duration of water and bitter substance consumption (Fig 1A and 1B, Table 1).

**Table 1:** RNAi lines used for the GABAergic receptor screen.

Name in Fig 1B	Stock #
*UAS-empty attp2*	Bloomington stock # 36303
*UAS-empty attp40*	Bloomington stock # 36304
*UAS-GABA-B-R1*	Bloomington stock # 28353
*UAS-GABA-B-R2*	Bloomington stock # 27699
*UAS-GABA-B-R3*	Bloomington stock # 42752
*UAS-GABA-B-R3(2)*	Bloomington stock # 26729
*UAS-Rdl(1)*	Bloomington stock # 31286
*UAS-Rdl(2)*	Bloomington stock # 31662
*UAS-Rdl-RNAi (3)*	Vienna# 41103
*UAS-Rdl-RNAi (4)*	Vienna 100429
*UAS-LCCH3*	Bloomington stock # 32019
*UAS-GRD-shRNA*	Bloomington stock # 38384
*UAS-GRD*	Bloomington stock # 29589

Wild-type flies that are hydrated and fed do not drink water or the bitter compound denatonium[17](Fig 1C and 1D), whereas flies with inactivated DSOG1 neurons consume a vast amount of water or denatonium[17]. As expected, control flies did not consume water or denatonium, (when they were hydrated and fed). In contrast, fed and hydrated flies with *Rdl* knocked down using two different RNA interference lines (*Rdl(3)* and *Rdl (4)*) that target different sequences of *Rdl*, significantly overconsumed both water and denatonium (Fig 1B), similar to the DSOG1 phenotype. Knocking down *Rdl* using the other two Rdl lines (1 and 2) did not result in a significantly different consumption time of water and bitter compared to all other lines. These behavioral differences likely reflect differences in the ability of different RNAi lines to effectively reduce gene expression. In contrast, knockdown of other GABAergic receptors resulted in little or no consumption of water or denatonium. Overconsumption of denatonium and water indicates that the drive to consume in these flies overrides normal bitter taste inhibition of feeding and water satiation, respectively. This suggests that the RDL receptor is the only GABAergic receptor required to restrict consumption of water and bitter substances in fed states.

Although RDL plays an important role in regulating consumption, it is broadly expressed in the nervous system and is not specific for neural circuits that mediate consumption. To screen for candidate RDL neurons that regulate consumption, we knocked down *Rdl*, using the line with the most robust overconsumption phenotype, *UAS-Rdl(3)*, in small subsets of neurons within the central nervous system, using existing Gal4 lines from the Clandinin, Flylight, and Vienna collections[23–26] and assayed for increased consumption of water or bitter in fed flies. Water consumption was variable, either because many neural subsets require RDL for water consumption or because of differences in genetic background. Nevertheless, RDL knockdown in neurons downstream of DSOG1 would be expected to have defects in both bitter and water consumption. Therefore, we selected lines that showed increased water and bitter consumption compared to wild-type and tested for reproducibility by comparing consumption time in fed flies with RDL knockdown to sibling controls (S1 Fig). Out of 183 Gal4 lines, we identified two Gal4 lines, *VT16839-Gal4* and *VT27941-Gal4* that showed robust and reproducible overconsumption of both denatonium and water compared to all other lines in the screen (Fig 1C and 1D and S1 Fig). Interestingly, the enhancer segment of VT27941 is a sequence mapping to the intronic region of *Rdl*.

**Fig 2:**
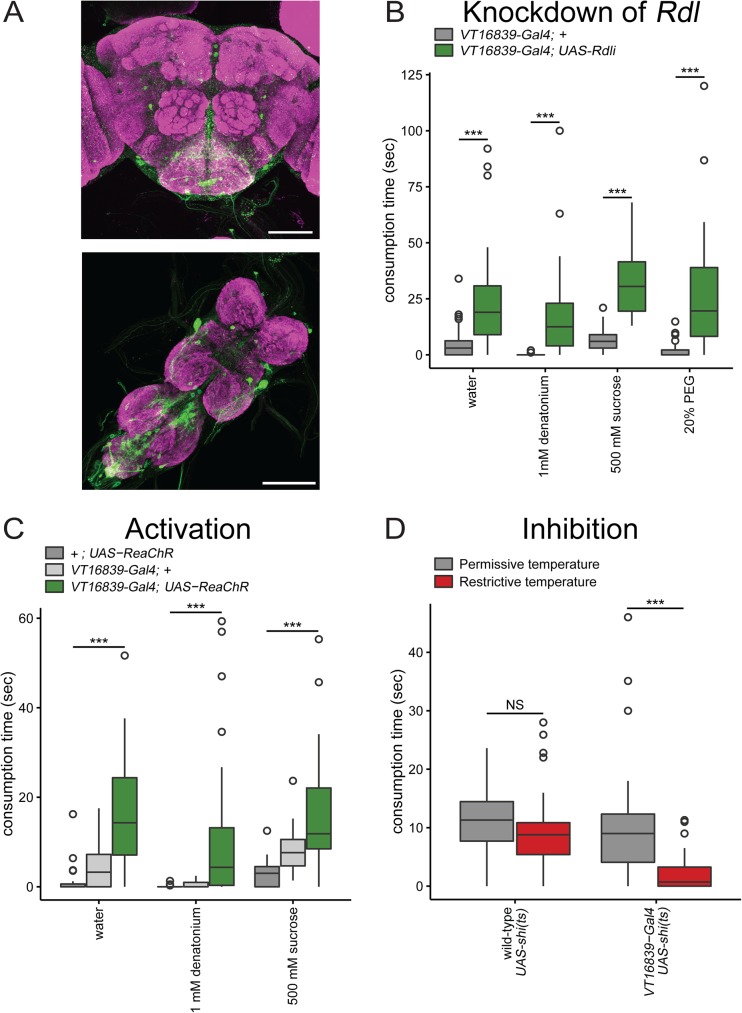
*VT16839-Gal4* neurons trigger and are critical in consumption behavior. **A)** *VT16839-Gal4* drives expression of *UAS-mCD8*::*GFP* in the brain and VNC (scale bar = 100 μm) **B)** RNA interference knockdown of *Rdl* in *VT16839-Gal4* neurons increased consumption time of water, 1 mM denatonium, 500 mM sucrose and 20% PEG. Box plot shows fed *UAS-Rdli; UAS-Dcr2/VT16839-Gal4* flies spent more time consuming water, 1 mM denatonium, and 500 mM sucrose compared to fed +; *UAS-Dcr2/VT16839-Gal4* flies Wilcoxon rank-sum test with continuity correction; ***p<0.0001; n = 22-72/genotype **C)** Activation of *VT16839-Gal4* neurons using ReaChR caused increased consumption time of appetitive (500 mM sucrose or water) and non-appetitive (1 mM denatonium) substances. Box plot shows fed *VT16839-Gal4; UAS-ReaChR* flies consumed more appetitive and non-appetitive substances compared to fed *VT16839-Gal4* and *UAS-ReaChR* flies; Kruskal-Wallis test, Dunn’s post-hoc; ***p<0.0001; n = 46-49/genotype **D)** Silencing of *VT16839-Gal4* neurons using *shibire*^*ts*^ *(shi(ts))* decreased consumption of sucrose (500 mM) in flies starved for 24 hours. Box plot shows 24 hour starved *W1118(wild-type); UAS-shi(ts)* spent more time consuming 500 mM sucrose compared to starved *VT16839-Gal4* /*UAS-shi(ts)* flies; Wilcoxon rank-sum test with continuity correction; ***p<0.0001; NS = not significant; n = 50-55/genotype; Permissive temperature = 19–21°, restrictive temperature = 30–33°

To determine which neurons are contained in the Gal4 lines, we crossed them to *UAS-mCD8*::*GFP* reporter flies and examined GFP expression in the brain and ventral nerve cord. Expression of Gal4-driven mCD8::GFP in *VT27941-Gal4* was broad throughout the central nervous system (data not shown), whereas the expression in *VT16839-Gal4* was sparse. *VT16839-Gal4* labels ~50 neurons in the brain and ~50 neurons in the ventral nerve cord (Fig 2A). We therefore focused on *VT16839-Gal4* for further study.

### *Rdl* expression in VT16839 neurons regulate consumption

The phenotype of DSOG1-mediated overconsumption is independent of taste quality or nutritional state[17]. To test whether knockdown of *Rdl* in VT16839 neurons also causes overconsumption of nutritive and non-nutritive substances, we used RNA interference and assayed consumption. Knocking down *Rdl* in *VT16839-Gal4* neurons resulted in fed flies that overconsumed denatonium, water, and sucrose compared to control flies (Fig 2B). These results suggest that levels of *Rdl* are critical for limiting ingestion of non-appetitive and appetitive tastants. Since *Rdl* in VT16839 neurons is essential to reduce consumption of nutritive and non-nutritive taste compounds, we wondered if it was also necessary to prevent consumption of compounds not detected by the gustatory system. We tested if the tasteless compound, 20% polyethylene glycol (PEG) was sufficient to drive overconsumption. 20% PEG is tasteless because its high osmolarity inhibits water-sensing neurons [5], and it is not detected by bitter and sweet sensory cells. Fed flies with *Rdl* knocked down in VT16839 neurons consumed significantly more 20% PEG compared to controls (Fig 2B), suggesting that the overconsumption phenotype is independent of taste inputs from sugar, bitter, or water taste neurons. Interestingly, overconsumption of 20% PEG was comparable to overconsumption of water, denatonium, and sucrose (Fig 2B). These results demonstrate that flies overconsume independent of taste quality when *Rdl* is knocked down in *VT16839-Gal4* neurons, indicating that GABA signaling through the RDL receptor acts to limit consumption under normal conditions.

### VT16839 neurons are necessary and sufficient for consumption

Knockdown of the GABA receptor, *Rdl*, in VT16839 neurons increased consumption, likely by decreasing inhibition and thus increasing activity of VT16839 neurons. Therefore, we hypothesized that directly increasing activity in VT16839 neurons might also promote consumption behavior. To test this, we acutely activated VT16839 neurons using *UAS-ReaChR*, an optically-gated non-specific cation channel [27]. We tested if these neurons could trigger consumption of tastants upon activation with 635 nm light. Activation of VT16839 neurons in fed flies resulted in overconsumption of not only sucrose and water but also denatonium (Fig 2C). In contrast, control flies that did not express *UAS-ReaChR* or *VT16839-Gal4*, showed little consumption. This argues that neurons in the *VT16839-Gal4 line* drive consumption likely downstream of taste quality checkpoints.

We tested whether VT16839 neurons are necessary for consumption by acutely silencing these neurons in starved flies, conditions where the fly had a strong drive to consume. If VT16839 neurons are necessary for consumption, we would expect to see a reduction in consumption when VT16839 neurons are silenced. We acutely silenced VT16839 neurons in starved flies using the temperature sensitive, dominant-negative dynamin, shibire^ts^ [28] which prevents synaptic vesicle recycling at 33°C but not at 22°C. Starved flies with VT16839 neurons silenced significantly reduced consumption of 500 mM sucrose compared to room temperature controls (Fig 2D). Taken together, these results demonstrate that VT16839 neurons are necessary and sufficient for consumption behavior.

### VT16839 neurons are sufficient to suppress DSOG1 mediated overconsumption

**Fig 3:**
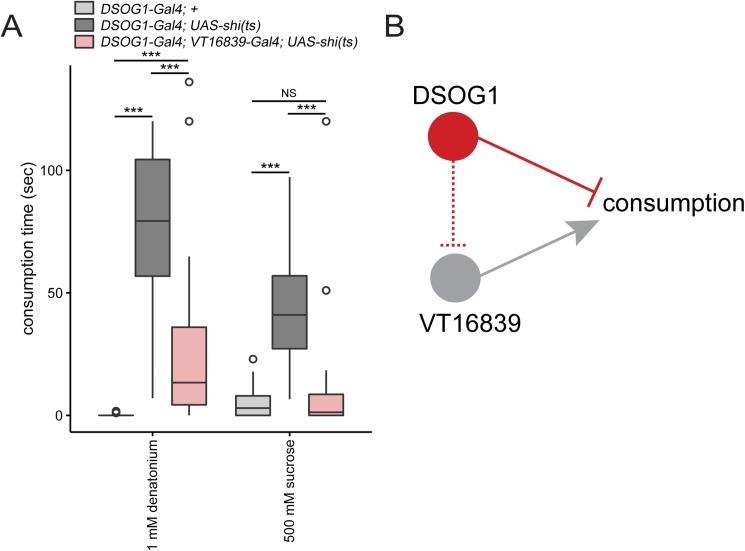
*VT16839-Gal4* overrides DSOG1-mediated overconsumption. **A)** Epistasis experiment. Co-silencing DSOG1 neurons *(98-Gal4)* and *VT16839-Gal4* neurons in fed flies decreased consumption time. Box plot shows *98-Gal4; UAS-shi(ts)/16839-Gal4* fed flies spent less time consuming 1 mM denatonium and 500 mM sucrose compared to *98-Gal4; UAS-shi(ts)* (DSOG1 neurons silenced) and *98-Gal4* fed flies. Kruskal-Wallis test, Dunn’s post-hoc; ***p<0.0001; n = 19-47/genotype **B)** Model. Silencing *VT16839-Gal4* overrides the overconsumption phenotype caused from silencing DSOG1 neurons.

DSOG1 neurons are GABAergic interneurons that inhibit consumption. VT16839 neurons are GABA-receptor expressing neurons that trigger consumption and are necessary for normal consumption. One hypothesis is that GABA release from DSOG1 binds to *Rdl* on VT16839 neurons to inhibit their activity and decrease consumption. To test whether VT16839 neurons are downstream of DSOG1 we performed an epistasis experiment by co-silencing the two populations of neurons. As expected, acutely silencing DSOG1 neurons in fed flies, using the *98-Gal4* to drive expression of shibire^ts^, results in overconsumption of both appetitive and aversive substances ([17], Fig 3A). As VT16839 neurons might be downstream of DSOG1, we tested whether blocking synaptic transmission in VT16839 neurons would suppress the overconsumption phenotype. We co-silenced DSOG1 and VT16839 neurons in fed flies using *UAS-shibire*^*ts*^ and measured consumption. Fed flies with DSOG1 and VT16839 neurons co-silenced consumed significantly less sucrose compared to DSOG1 silenced flies but did not consume significantly different from DSOG1 genetic control *(98-Gal4)* (Fig 3A). In addition, co-silencing both populations of neurons also resulted in a significant suppression of the DSOG1-mediated consumption of denatonium. Co-silencing both populations resulted in significantly more denatonium consumption compared to the DSOG1 genetic control, which may be because the strength of the VT16839 driver may not be strong enough to completely overcome DSOG1-mediated denatonium consumption. These results demonstrate that silencing VT16839 neurons can override overconsumption caused by inhibition of DSOG1 neurons. These data are consistent with the notion that VT16839 neurons act downstream of DSOG1 neurons (Fig 3B), although they do not rule out the possibility of parallel pathways.

### A Subpopulation of VT16839 neurons trigger consumption

**Fig 4:**
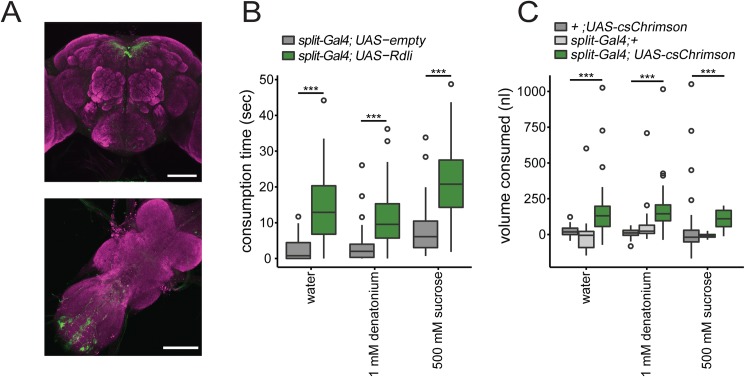
A subset of *VT16839-Gal4* neurons trigger consumption. **A)** *VT16839-Gal4-DBD*, *VT27941-Gal4-AD* flies drives expression of *UAS-mCD8*::*GFP* in the brain and VNC (scale bar = 100 μm) **B)** Knockdown of *Rdl* in VT16839-split neurons resulted in increased consumption time. Box plot shows fed *UAS-Rdli; UAS-Dcr2/VT16839-split-Gal4(split-Gal4)* flies spent more time consuming water, 1 mM denatonium, and 500 mM sucrose, compared to *UAS-empty attp40*; *UAS-Dcr2/VT16839-split-Gal4flies (split-Gal4)* Wilcoxon rank-sum test with continuity correction; ***p<0.0001; n = 64-70/genotype **C)** Activation of *VT16839-Gal4* neurons using csChrimson increased consumption volume of appetitive (500 mM sucrose or water) and non-appetitive (1 mM denatonium) substances. Box plot shows fed *VT16839-split-Gal4(split-Gal4); UAS-csChrimson* flies consume more volume of appetitive and non-appetitive substances; Kruskal-Wallis test, Dunn’s post-hoc; ***p<0.0001; n = 21-63/genotype

We sought to narrow down the causal neurons in *VT16839-Gal4* that trigger consumption by using an intersectional approach to limit expression to subsets of VT16839 neurons. Because knocking down *Rdl* in either *VT16839-Gal4* or *VT27941-Gal4* resulted in overconsumption of water and denatonium, we decided to look for neurons present in both Gal4 lines. We made split-Gal4 driver lines where the promoter sequence of each Gal4 line was used to drive expression of a complementary half of Gal4 (*VT16839-split-Gal4)*. Only neurons that have both halves of the Gal4 will reconstitute a functional Gal4 to drive expression of effector proteins. Therefore, this approach limits functional Gal4 expression to the neurons common to both driver lines [29]. As a result, functional Gal4 expression of mCD8::GFP was limited to ~10 neurons in the brain and ~16 in the VNC (Fig 4A). In the brain, there was a class of neurons strongly labeled in higher brain whose projections span near the pars intercerebralis along with several dimly labeled neurons in the SEZ. An additional two populations were labeled in the abdominal segment of the VNC.

We tested whether the *VT16839-split-Gal4* driver lines do indeed contain neurons that trigger consumption by knocking down *Rdl* using RNAi. Knockdown of *Rdl* in the *VT16839-split-Gal4* resulted in overconsumption of both appetitive and aversive stimuli, indicating that neurons that promote consumption are labeled by the *VT16839-split-Gal4* (Fig 4B). Similarly, we tested whether the neurons labeled by the *VT16839-split-Gal4* can trigger consumption through acute activation experiments. Since activation using ReaChR resulted only in a small increase in consumption (S2 Fig), we used an alternative optically-gated cation channel, csChrimson [30] to activate the neurons upon red light exposure and measured consumption time. Activation caused the majority of flies to constitutively pump their proboscis (45 of 51 flies) in the absence of any stimulus. The constitutive pumping behavior may indicate a drive to consume that does not require a stimulus, suggesting that csChrimson strongly activates the neurons.

In the csChrimson experiments, consumption was masked by constitutive pumping, making it difficult to reliably measure consumption time. As a result, we decided to quantify consumption by measuring volume ingested instead of time spent drinking. We estimated volumes of ingestion by utilizing capillary tubes and measuring the volumes before and after individual flies drank. When *VT16839-split-Gal4* neurons were activated using csChrimson, fed flies consumed more appetitive and aversive tastants in our volumetric consumption assay (Fig 4C) compared to control fed flies. This indicates that *VT16839-split-Gal4* neurons trigger consumption of both appetitive and non-appetitive substances. The *VT16839-split-Gal4* thus labels a small number of GABAergic receptor neurons that promote consumption. Furthermore, these RDL expressing neurons promote consumption independent of taste inputs.

## Discussion

The dissection of neural circuits that underlie consumption remains an important challenge toward understanding the regulation of feeding behavior. Our study identifies neurons that regulate the consumption of non-appetitive and appetitive substances, and depend on the expression of RDL receptor for proper regulation of consumption. These RDL receptor-expressing neurons are able to orchestrate consumption regardless of taste quality, as knockdown of *Rdl* expression within these neurons not only causes overconsumption of sugar, bitter, and water substances, but tasteless substances as well. Acute activation of these neurons also caused overconsumption of sweet, bitter and water substances, whereas blocking neurotransmission of these neurons results in decreased sucrose consumption in starved flies. These studies reveal a subset of neurons that play a critical role in promoting consumption.

Previous studies have identified two different classes of interneurons that trigger sucrose consumption. FDG neurons are located in the SEZ and respond to sugar stimulation on the proboscis [10] and the cholinergic IN1 neurons respond to sugar stimulation of the internal mouthparts [11]. These two classes of neurons respond selectively to sucrose, suggesting that there is a pathway selective for regulating sucrose consumption. Similarly, ectopic activation of these neurons increased consumption of sucrose but not water or bitter. These studies indicate that consumption of sucrose is regulated independently of consumption of water or bitter and argue for distinct circuits mediating consumption for each class of tastant. The RDL-expressing neurons differ from previously identified consumption neurons because either knockdown of *Rdl* or optogenetic activation of these neurons elicited consumption not only of appetitive substances, but also of non-appetitive substances. One model suggested by these studies that bears testing is that there may be distinct circuits for sweet, water, and bitter food sources that all converge on the RDL-expressing neurons.

Knockdown of *Rdl* results in increased consumption of water, sucrose and bitter substances. These RDL neurons may be inhibited by GABAergic neurons such as DSOG1. Previous studies indicate that DSOG1 neurons act as a tonic inhibitor of consumption[17]. Flies with silenced DSOG1 neurons overconsume all taste substances independent of taste quality and nutritional state, very similar to the phenotype observed when activating the RDL neurons in this study. An attractive model is that GABA release from DSOG1 inhibits the RDL neurons, restricting consumption. Indeed, our studies show that RDL neuronal silencing is able to suppress the DSOG1-silencing phenotype. Although our data are consistent with the model that DSOG1 acts on the RDL neurons, it remains possible that the RDL neurons and DSOG1 influence parallel pathways. Further characterization of the RDL neurons that promote consumption and the DSOG1 neurons that inhibit consumption will enable us to distinguish these models.

Together, our study demonstrates that RDL function in a subset of neurons is critical for the regulation of consumption of all substances, regardless of taste modality. Further studies characterizing these neurons and their interactions with the different neurons that regulate feeding will provide insight into the temporal dynamics and plasticity in feeding decisions.

## Materials and methods

### Experimental animals

The Gal4 lines used for the behavioral screens were from the following collections: the Janelia Flylight collection [23], Vienna VT collection [25,26] and the Clandinin Gal4 collection [24]. GABAergic receptor RNAi lines were from the TRiP and VDRC collection [22]. The following fly lines were used: *nSyb-Gal4* [21], *UAS-ReachR* (II) [27], *UAS-csChrimson* (X) [30], *UAS-Shibire*^*ts*^ [28]. *UAS- Dicer2 (III)* (BDSC #24651) was used in all RNA interference experiments. *98-Gal4* is from the Clandinin Gal4 collection, while *VT16839-Gal4* and *VT27941-Gal4* are from the Vienna VT collection.

For *VT16839-split-Gal4*: *VT16839-Gal4* and *VT27941-Gal4* enhancer segments were amplified using genomic DNA from IsoD1 flies. *VT16839-Gal4* and *VT27941-Gal4* enhancer segments were cloned into pBPZpGAL4DBDUw (Addgene Plasmid #26233) plasmid and pBPp65ADZpUw (Addgene Plasmid #26234) plasmid, respectively. Cloned plasmid were injected into attp2 (*VT16839-Gal4-DBD*) and vk00027 sites (*VT27941-Gal4-AD*) (BestGene).

### Behavioral experiments

Female flies were tested for behavior 5–10 days after eclosion. All flies with the exception for *UAS-shibire*^*ts*^ flies (20°C) were grown at 25°C and flipped onto fresh food 2 days prior to the experiment. All starved experimental flies were flipped into vials with wet kimwipes for allotted starvation time. Flies were glued on slides with nail polish and then were placed 2–4 hours in a humidified chamber. Temporal consumption assays were performed as previously described [17]. Time spent consuming was recorded until flies rejected the substance ten times. For RNAi experiments, *UAS-Dicer2* (III) was used to boost efficiency of the knockdown. For Figs 1C, 1D, 2, 3 and 4, *UAS-Rdl-RNAi (3) (*Vienna# 41103) line was used to knock down *Rdl*.

For activation experiments using ReaChR and csChrimson, experimental flies were kept on 0.4 mM all-trans retinal (Sigma), 2–4 days prior to the experiment. Activation experiments were done under 635 nm light (LaserGlow) and assayed one fly at a time. Flies were habituated under the laser for 1–2 minute prior to assay. For shibire^ts^ experiments, mounted flies were incubated at 30–32°C on a peltier device and given water for 10–15 min prior to experiment.

For volumetric consumption assays, Drummond Wiretrol capillaries (CAT #5-000-1003) were filled with 5 μl of tastant mixed with blue dye (0.25mg/mL blue dye (Erioglaucine, Sigma)) and attached to tubing connected to a 1 ml syringe. One edge of the capillary was painted with vasoline to prevent spilling of tastant. Before and after pictures were taken in the presence of a standard ruler to correct for zoom. Difference in pixels before and after was measured using Photoshop. A standard pixel-to-volume conversion factor was calculated by pipetting volumes into various pipettes and creating a graph with a linear fit (S3 Fig). Volume was calculated by converting the pixels to mm lost before and after behavioral assay based on the standard pixel-to volume conversion factor.

### Statistics

Data from Figs 1, 2, 3 and 4 are all non-parametric, therefore non-parametric statistical tests were used. When multiple samples were being compared, Kruskal-Wallis test with Dunn’s post-hoc was used (Figs 1B, 2C, 3A and 4C). When two samples were being compared, Wilcox rank-sum test was used with continuity correction (Figs 2B, 2D and 4B, and S1 and S2 Figs).

### Immunohistochemistry

Antibody fixation and staining was performed as previously described[17].The following antibodies were used: rabbit anti-GFP (Invitrogen, 1:1000); mouse anti-nc82 (Hybriodoma bank 1:500). The following secondary antibodies were used (Invitrogen at 1:100): 488 anti-rabbit, 568 anti-mouse. All images were taken using a Zeiss confocal microscope. Brightness and contrast was adjusted for images using ImageJ.

## Supporting information

S1 Fig *VT16839-Gal4* and *VT27941-Gal4* lines show increased water consumption with *Rdl* knockdown. Knockdown of *Rdl* in candidate Gal4 lines. Bar graph showing time consumption of water (mean ± SEM) in fed flies. Candidate Gal4 lines were tested against sibling flies (no RNAi) for reproducibility of overconsumption phenotype for water. Both *VT16839-Gal4* and *VT27941-Gal4* showed robust and reproducibility of overconsumption; Wilcoxon rank-sum test with continuity correction;**p<0.01;***p<0.0001 n = 11-35/genotype
(TIF)

S2 Fig Activation of *VT16839-split-Gal4* neurons result in a small increase in consumption. Bar plot shows (mean ± SEM) activation of *VT16839-split-Gal4* caused small increases in 1 mM denatonium and water consumption time in *UAS-ReaChR; VT16839-split-Gal4* fed flies compared to *VT16839-split-Gal4* fed flies. Wilcoxon rank-sum test with continuity correction; ***p<0.001; n = 27-31/genotype
(TIF)

S3 Fig Pixel-to-volume conversion factor in the volumetric consumption assay. Scatter plot shows the pixels to volume when standard volumes (5 ul and 1 ul) were pipetted into capillaries. A curve was fit linearly to find the factor (y = 0.0058x) to convert the pixels changes to volume changes.
(TIF)
