# Supplementary figures and images for: GABAA receptor-expressing neurons promote consumption in Drosophila melanogaster
Source: PLoS One. 2017 Mar 31;12(3):e0175177. doi: 10.1371/journal.pone.0175177 (PMC5376345; doi:10.1371/journal.pone.0175177)

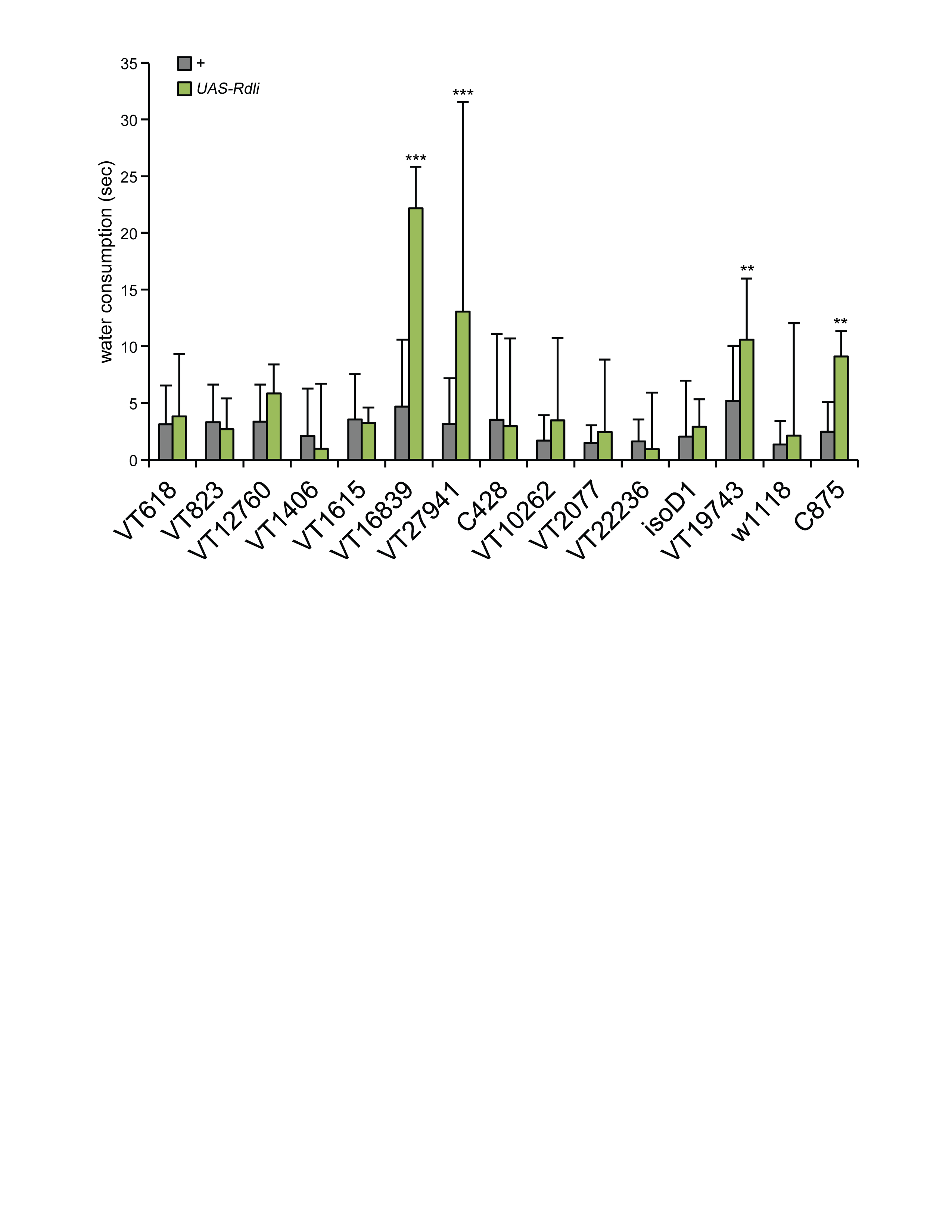

Supplement: S1 Fig — Knockdown of Rdl in candidate Gal4 lines. Bar graph showing time consumption of water (mean ± SEM) in fed flies. Candidate Gal4 lines were tested against sibling flies (no RNAi) for reproducibility of overconsumption phenotype for water. Both VT16839-Gal4 and VT27941-Gal4 showed robust and reproducibility of overconsumption; Wilcoxon rank-sum test with continuity correction;**p<0.01;***p<0.0001 n = 11-35/genotype (TIF) [file pone.0175177.s001.tif]

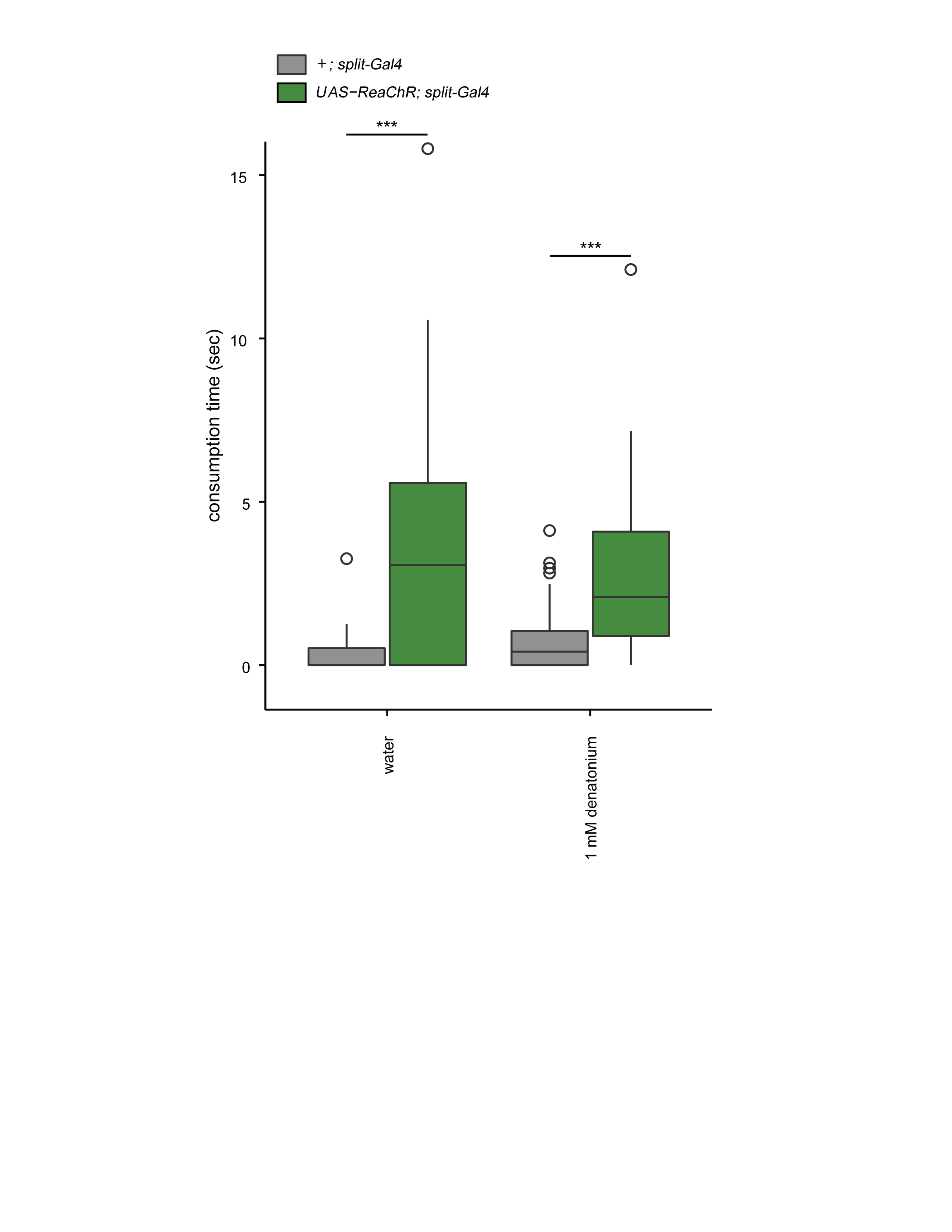

Supplement: S2 Fig — Bar plot shows (mean ± SEM) activation of VT16839-split-Gal4 caused small increases in 1 mM denatonium and water consumption time in UAS-ReaChR; VT16839-split-Gal4 fed flies compared to VT16839-split-Gal4 fed flies. Wilcoxon rank-sum test with continuity correction; ***p<0.001; n = 27-31/genotype (TIF) [file pone.0175177.s002.tif]

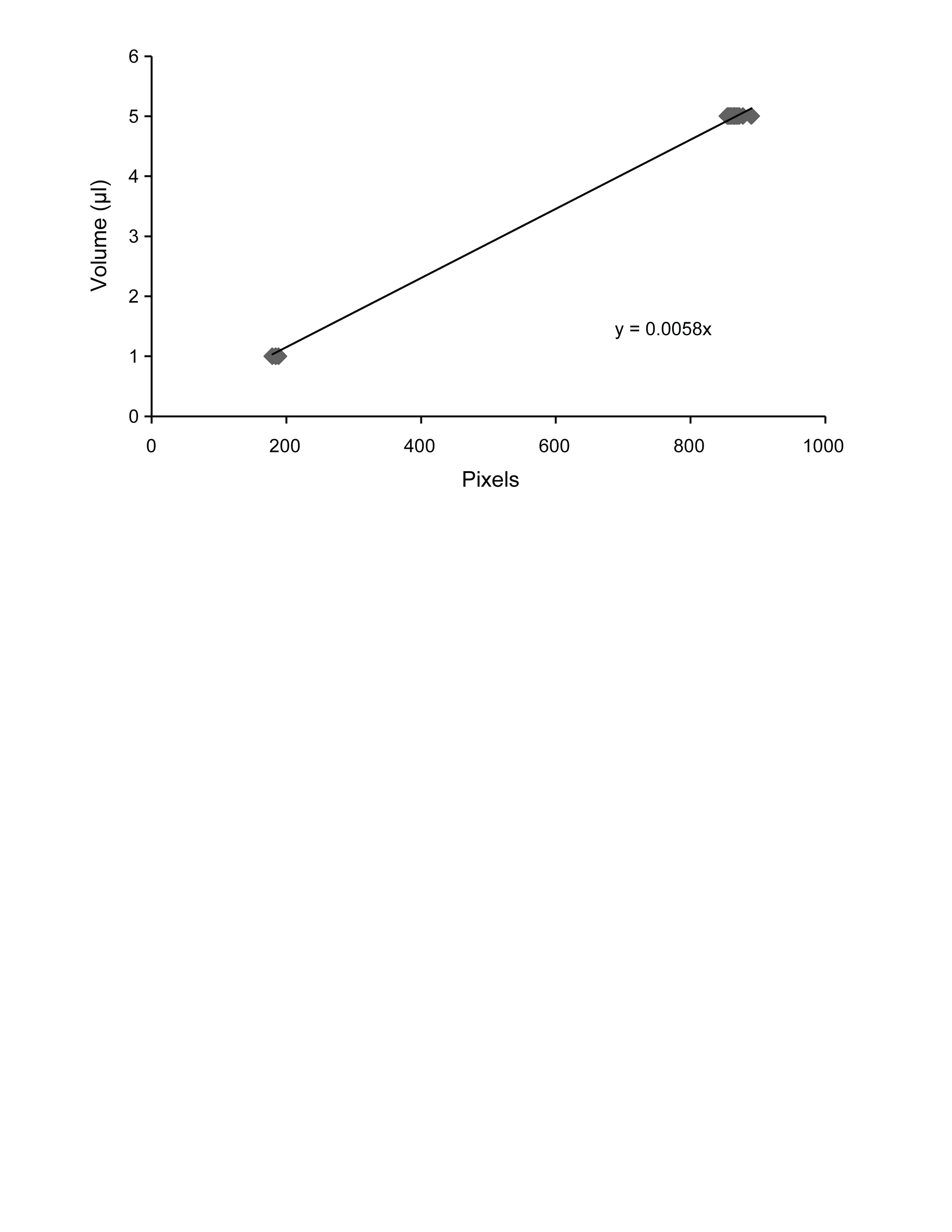

Supplement: S3 Fig — Scatter plot shows the pixels to volume when standard volumes (5 ul and 1 ul) were pipetted into capillaries. A curve was fit linearly to find the factor (y = 0.0058x) to convert the pixels changes to volume changes. (TIF) [file pone.0175177.s003.tif]
